# Supplementary material for: A Generic Multivariate Framework for the Integration of Microbiome Longitudinal Studies With Other Data Types
Source: Front Genet. 2019 Nov 7;10:963. doi: 10.3389/fgene.2019.00963 (PMC6875829; doi:10.3389/fgene.2019.00963)
Supplement: Supplementary file 1 [file DataSheet_1.pdf]

Supplemental material: A generic multivariate framework for the integration of microbiome longitudinal studies with other data types

Antoine Bodein [1†], Olivier Chapleur [2†], Arnaud Droit [1], Kim-Anh Lê Cao [3,*]

[1]CHU de Québec Research Center, Université Laval, Molecular Medicine department, Québec, QC, Canada,

[2] Hydrosystems and Bioprocesses Research Unit, Irstea, Antony , France,

[3]Melbourne Integrative Genomics, School of Mathematics and Statistics, University of Melbourne, Melbourne, VIC, Australia

[†] Both authors contributed equally to this manuscript

1 Linear Mixed Model Splines (LMMS) models

The first model assumes the response is a straight line not affected by individual variation. Let $y_{ij}(t_{ij})$ be the taxa normalized count for individual (or biological replicate) i at time t_{ij}, where $i = 1, 2, ..., n$, $j = 1, 2, ..., m_i$, N is the sample size and m_i is the number of observations for individual i for the given taxa. A simple linear regression of abundance $y_{ij}(t_{ij})$ on time t_{ij}, with the intercept β_0 and slope β_1 is estimated via ordinary least squares:

$$y_{ij}(t_{ij}) = \beta_0 + \beta_1 t_{ij} + \epsilon_{ij}, \quad \text{where} \quad \epsilon_{ij} \quad \sim \quad N(0, \sigma_\epsilon^2). \tag{1}$$

As nonlinear response patterns are commonly encountered, a second model uses a spline truncated line basis as proposed by Durban Durbán et al. (2005) to model a curve:

$$y_{ij}(t_{ij}) = f(t_{ij}) + \epsilon_{ij}, \quad \text{where} \quad \epsilon_{ij} \sim N(0, \sigma_\epsilon^2), \tag{2}$$

where f represents a penalized spline which depends on a set of knot positions $\kappa_1, ..., \kappa_K$ in the range of $\{t_{ij}\}$, some unknown coefficients u_k, an intercept β_0 and a slope β_1, i.e.

$$f(t_{ij}) = \beta_0 + \beta_1 t_{ij} + \sum_{k=1}^{K} u_k (t_{ij} - \kappa_k)_+,$$

$$\text{with} \quad (t_{ij} - \kappa_k)_+ = \begin{cases} t_{ij} - \kappa_k & \text{if} \quad t_{ij} - \kappa_k > 0, \\ 0 & \text{otherwise.} \end{cases}$$

The choice of the number of knots K and their positions influences the flexibility of the curve. As proposed by Ruppert (2002), we estimate the number of knots based on the number of measured time points T as $K = max(5, min(\lfloor \frac{T}{4} \rfloor, 40))$, placing the knots $\kappa_1 ... \kappa_K$ at quantiles of the time interval of interest.

A third model accounts for individual variation in Eq. (3) with the addition of a subject-specific random effect U_i to the mean response $f(t_{ij})$. We assume $f(t_{ij})$ to be a fixed (yet unknown) population curve, U_i is treated as a random realisation from an underlying Gaussian distribution independent from the previously defined random error ϵ_{ij}. The individual curves are expected to be parallel to the mean curve as we assume the subject-specific random effects to be constant over time:

$$y_{ij}(t_{ij}) = f(t_{ij}) + U_i + \epsilon_{ij}, \quad \text{where} \quad U_i \sim N(0, \sigma_U^2). \tag{3}$$

The final and fourth model is an extension to Eq. (3) that assumes individual deviations are straight lines, where individual-specific random intercepts a_{i0} and slopes a_{i1} are fitted:

$$\begin{aligned} &y_{ij}(t_{ij}) = f(t_{ij}) + a_{i0} + a_{i1} t_{ij} + \epsilon_{ij}, \\ &\text{with} \quad \epsilon_{ij} \quad \sim N(0, \sigma_\epsilon^2) \quad \text{and} \quad (a_{i0}, a_{i1})^T \sim N(0, \Sigma). \end{aligned} \tag{4}$$

*Corresponding Author: kimanh.lecao@unimelb.edu.au

Here we assume independence between the random intercept and slope, so the covariance matrix for the random effects Σ is diagonal.

2 Supplementary Figures

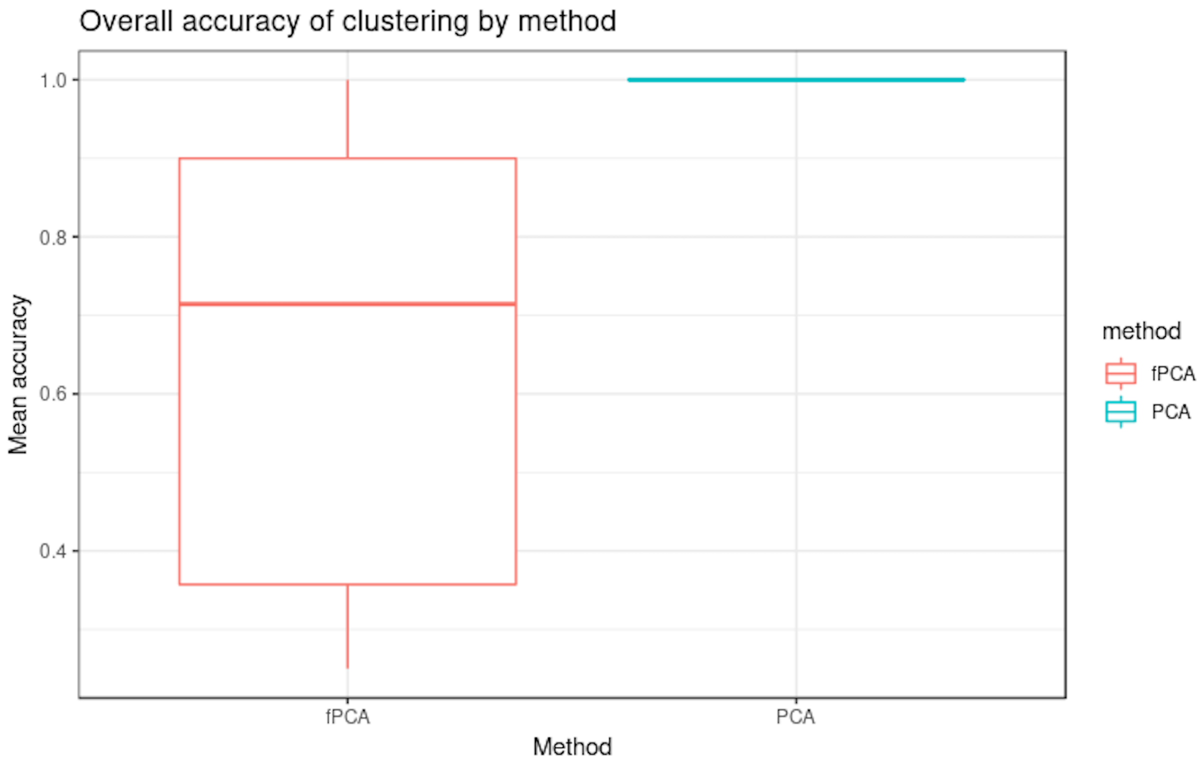

Figure 1: Simulation study: Overall accuracy of PCA and fPCA clustering with no noise. We compared the ability of PCA and fPCA to correctly assign the simulated profiles in their respective reference clusters. Without noise, fPCA clustering led to a poorer accuracy compared to PCA.

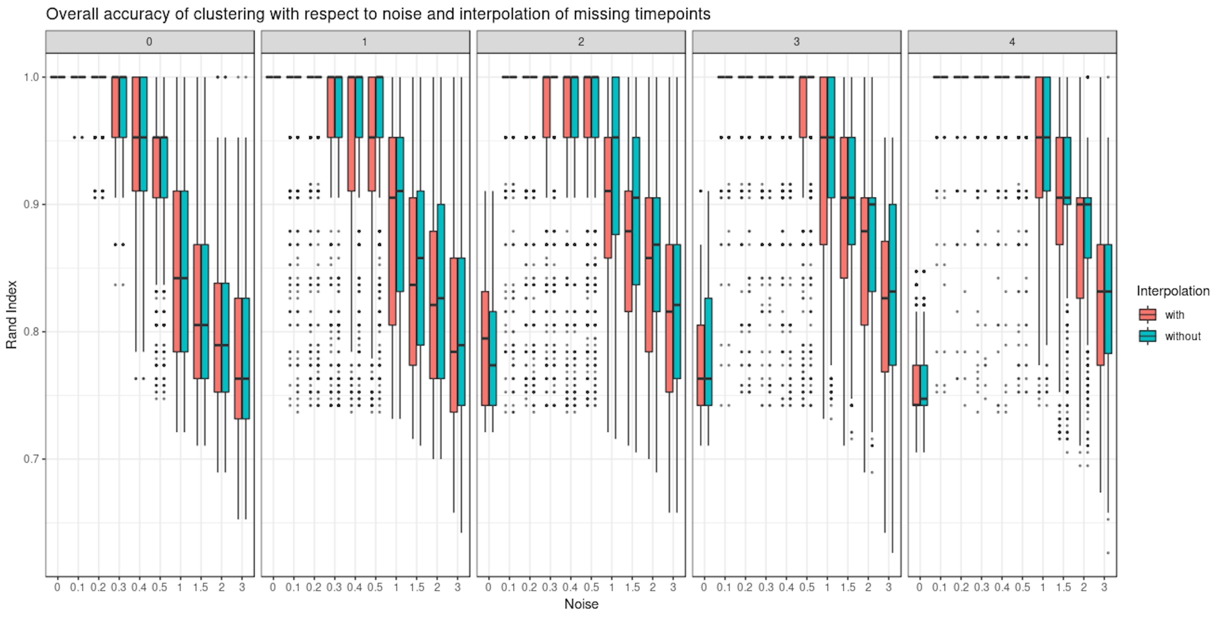

Figure 2: Simulated study: Overall compactness of assigned clusters when time points are missing with the Rand index.

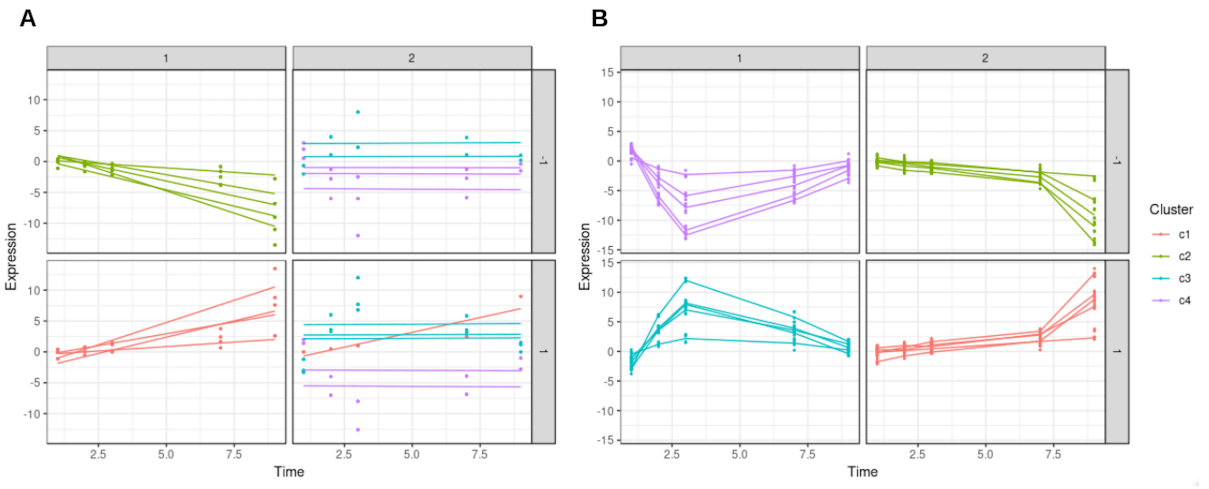

Figure 3: Simulation study: Clustering of simulated profiles with 4 missing time points, when time points are missing. Colors represent the ground truth cluster. (**A**) When noise = 0, LMMS mostly modelled straight lines, resulting in a poor clustering assignation. (**B**) When noise = 0.5, LMMS modelled splines resulted in better clustering.

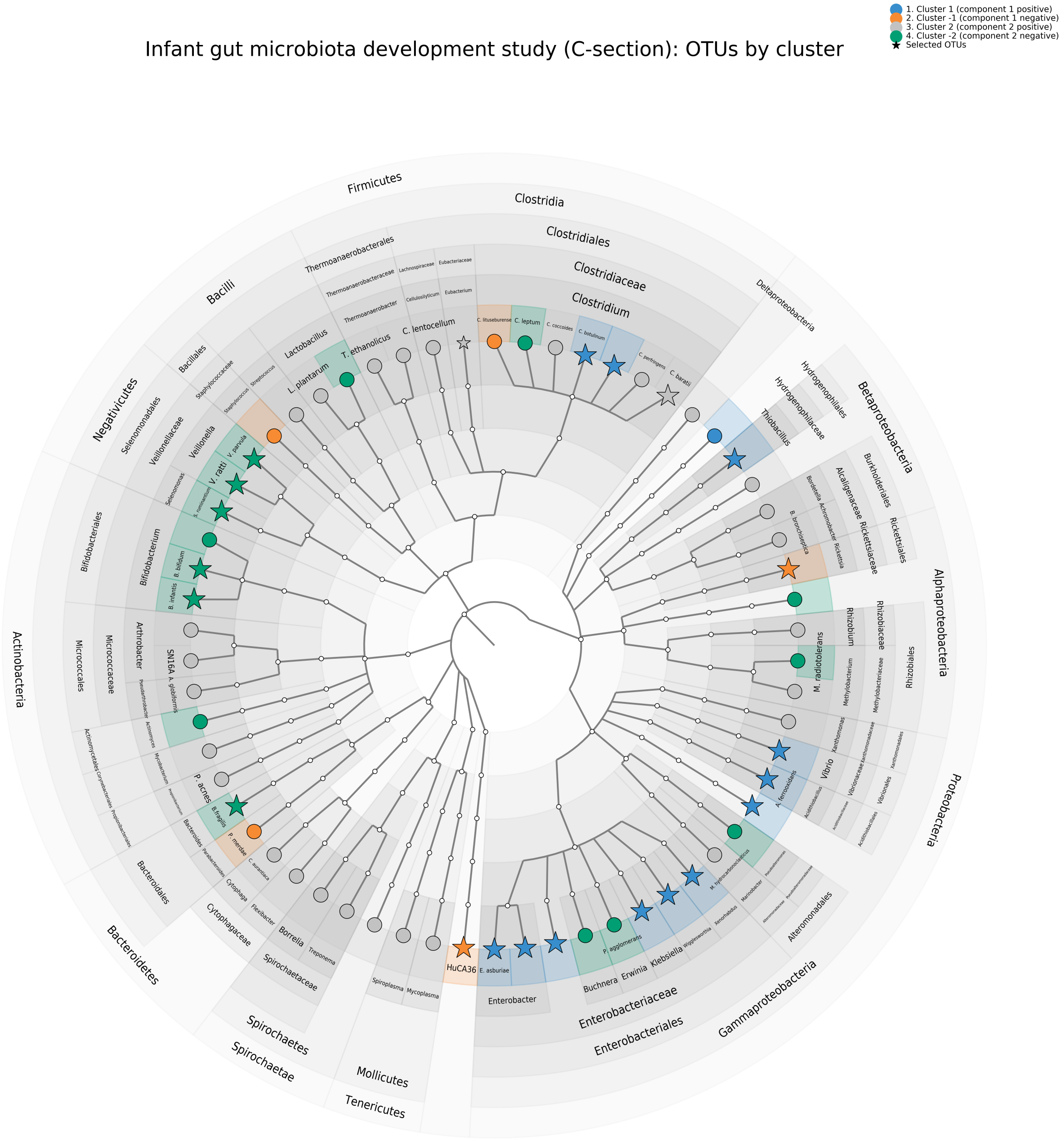

Figure 4: Infant gut microbiota development study, C-section data. Cladogram generated from PCA clustering result for C-section data using GraphlAn. Terminal nodes were colored according to the clustering, and OTUs selected with sPCA are represented with a star.

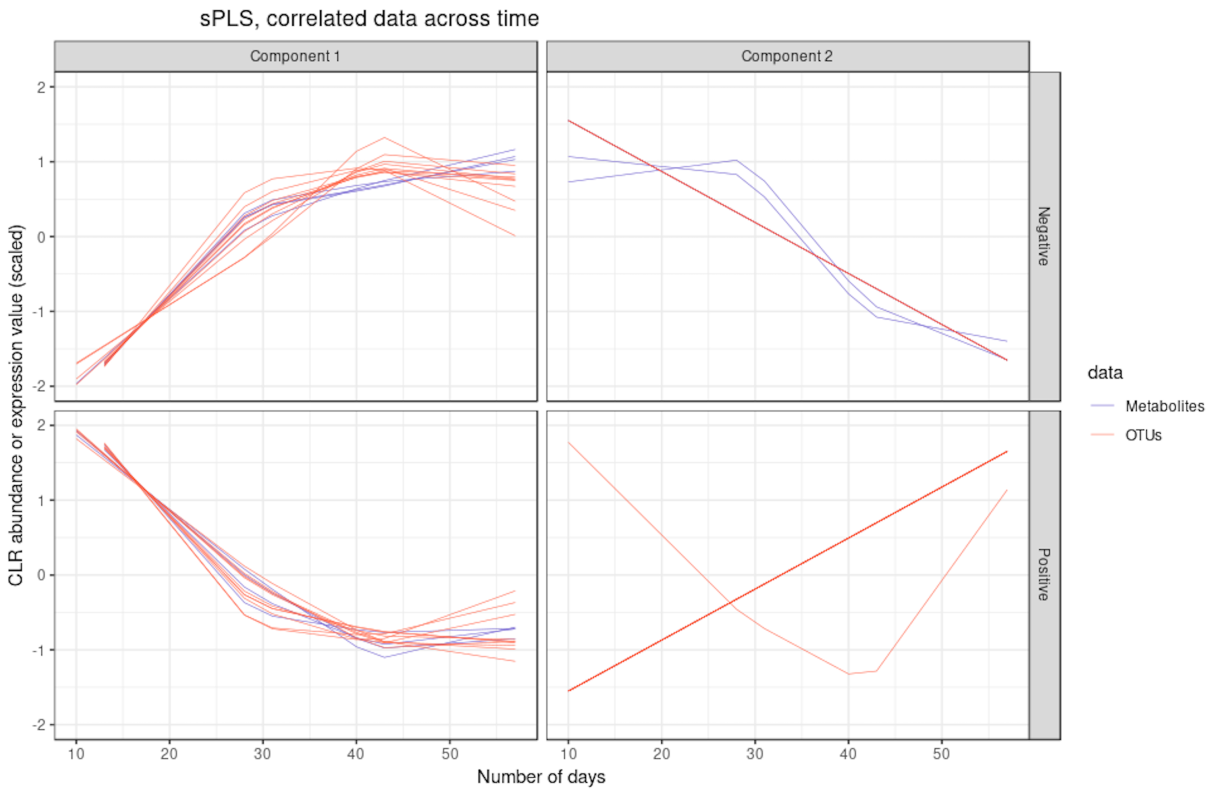

Figure 5: Waste degradation study: sPLS analysis identified subsets of associated OTUs and metabolites profiles. Each line represents the relative abundance of OTUs and metabolites selected by sPLS across time. OTUs and metabolites were clustered according to their contribution on each component. The clusters were further separated into profiles denoted ‘positive’ or ‘negative’ that refer to the sign of the loading vector from sPLS.

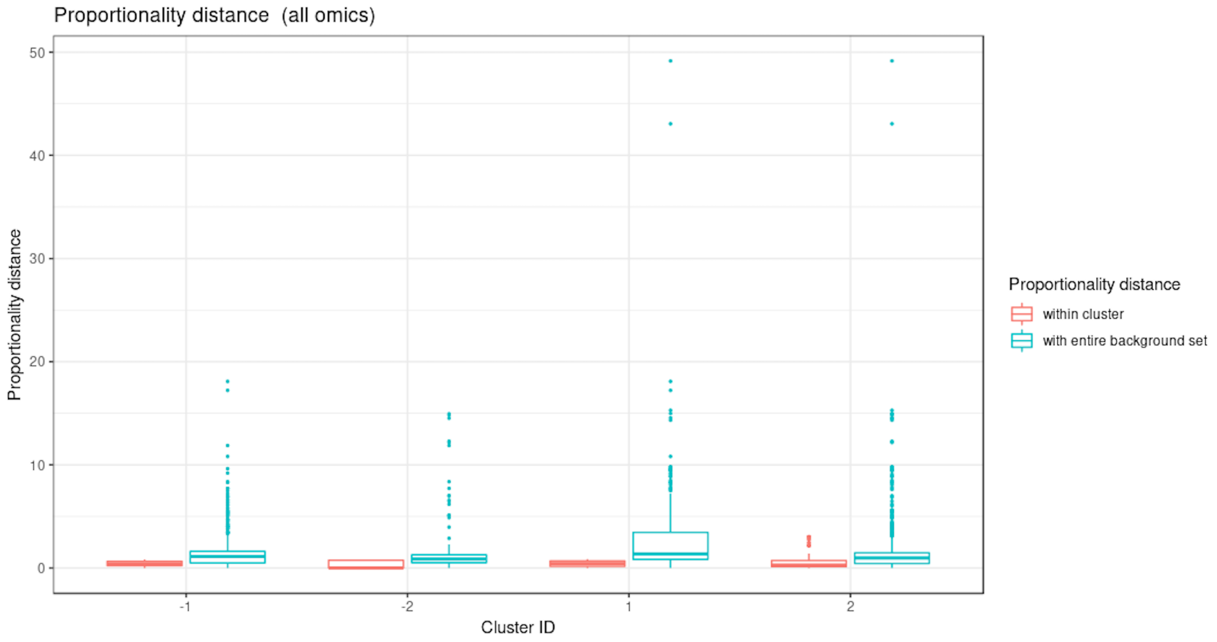

Figure 6: Waste degradation study: Proportionality distance per cluster identified with sparse PLS. The distance was calculated between each pair of profiles within a given cluster and with the entire background set (outside a given cluster).

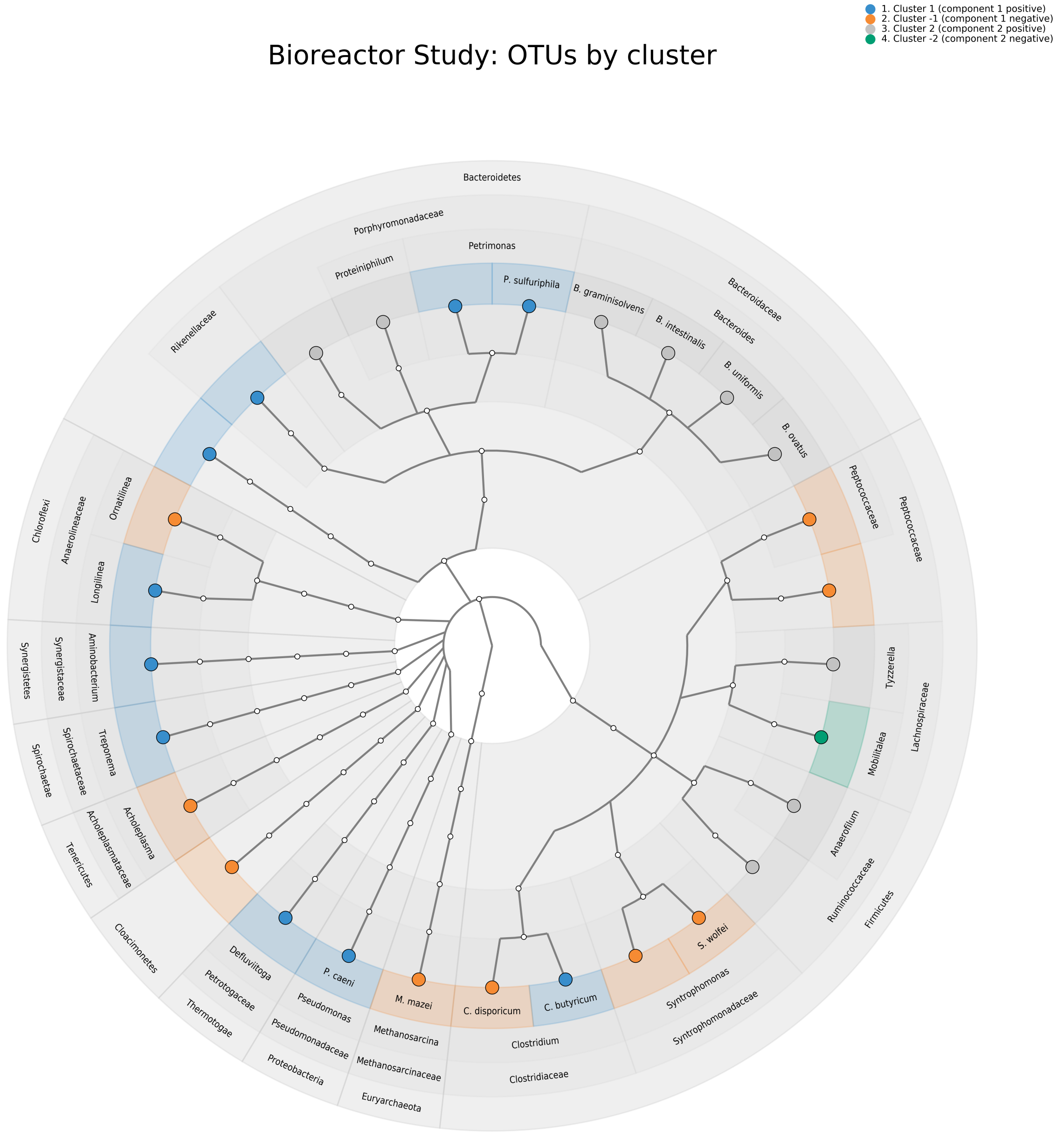

Figure 7: Waste degradation study. Cladogram generated from sparse PLS clustering result for data using GraphlAn. Only the selected OTUs are represented, with terminal nodes colored according to the clustering.

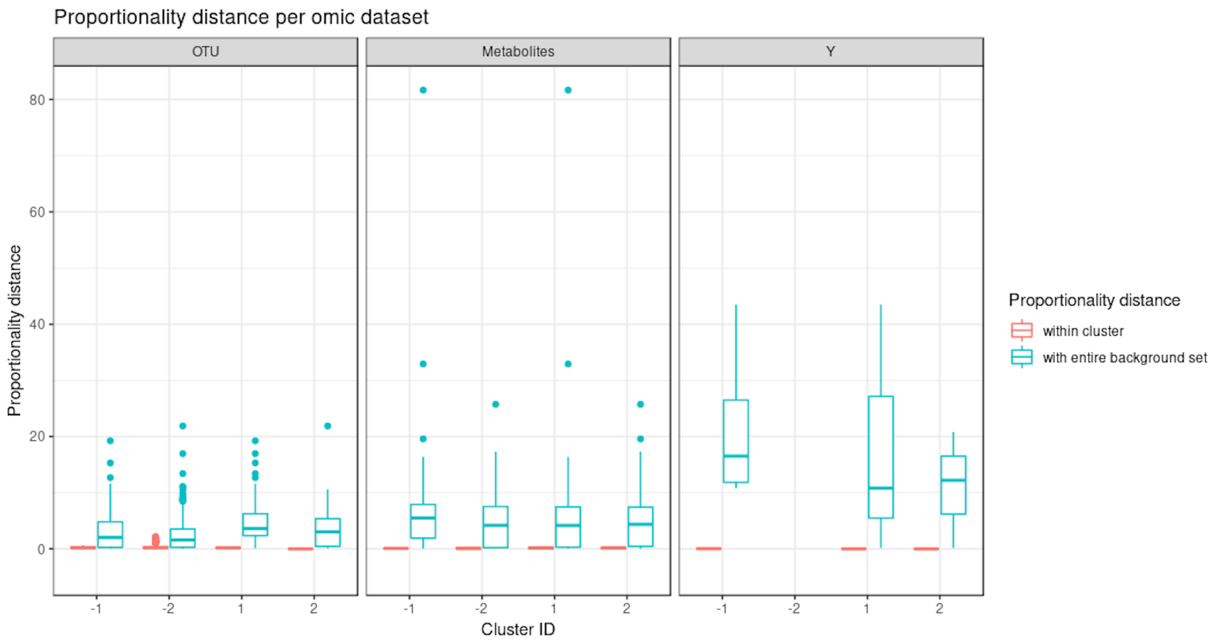

Figure 8: Waste degradation study: Proportionality distance per cluster and per omic dataset identified with multiblock sparse PLS clustering. The distance was calculated between each pair of profiles of the same type of omics data type within a given cluster and with the entire background set (outside a given cluster). Distances are displayed per omic dataset.

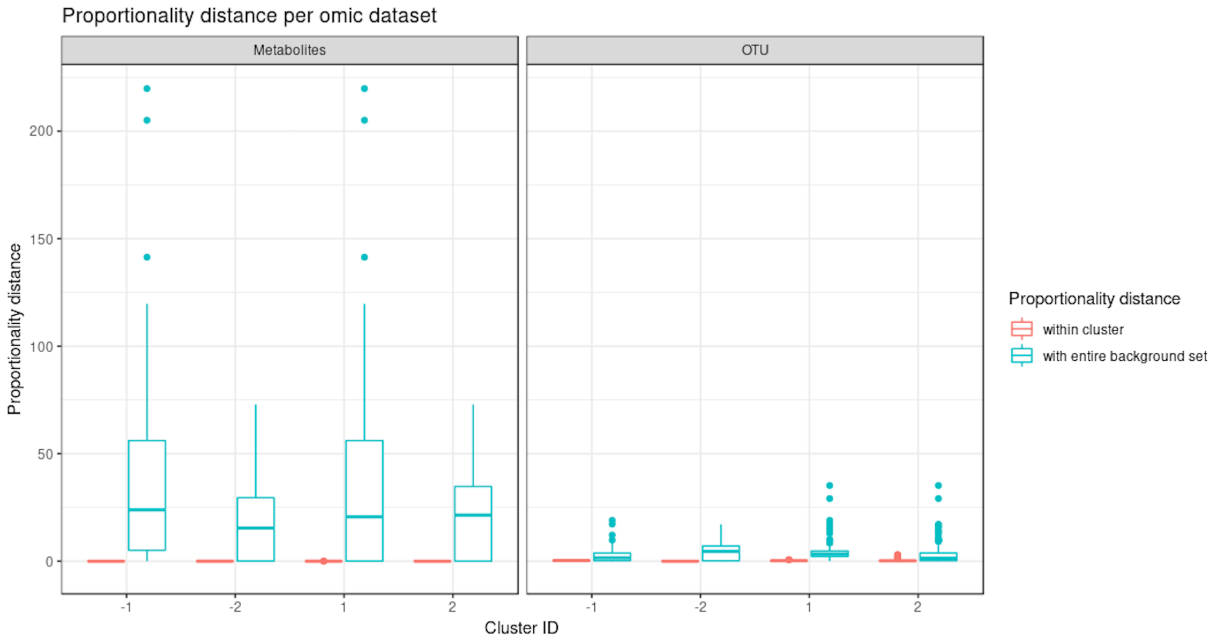

Figure 9: Waste degradation study: Proportionality distance per cluster and per omic dataset on the profiles identified with sparse PLS clustering. The distance was calculated between each pair of profiles of the same type of omics data type within a given cluster and with the entire background set (outside a given cluster). Distances are displayed per omic dataset.

3 Supplementary Figures

Table 1: Infant gut microbiota development for vaginal delivery. Proportionality distance for clusters identified with PCA. The median distance between all pairs of profiles, within cluster and with the entire background set (outside a given cluster) is reported. A Wilcoxon test p-value assesses the difference between the medians.

cluster	median within cluster	median outside cluster	Wilcoxon test P-value
1 (comp 1 positive)	0.64	1.3	$2.99 * 10^{-149}$
-1 (comp 1 negative)	0.2	1.44	$\leq 10^{-149}$
2 (comp 2 positive)	0.44	1.11	$2.59 * 10^{-15}$
-2 (comp 1 negative)	0.35	0.95	$2.75 * 10^{-19}$

Table 2: Infant gut microbiota development study: Proportionality distance between all pairs of OTUs. Clustering assignments from PCA and sPCA are indicated, along with whether the OTU was selected with sPCA. First sheet: vaginal data, Second sheet: for C-section data. External file 'Table1.xls'.

Table 3: Infant gut microbiota development for C-section delivery. Proportionality distance for clusters identified with PCA. The median distance between all pairs of profiles, within cluster and with the entire background set (outside a given cluster) is reported. A Wilcoxon test p-value assesses the difference between the medians.

cluster	median within cluster	median outside cluster	Wilcoxon test P-value
1 (comp 1 positive)	0.11	1.36	$3.46*10^{-80}$
-1 (comp 1 negative)	0.29	0.96	$1.36*10^{-15}$
2 (comp 2 positive)	0.67	1.5	$1.02*10^{-103}$
-2 (comp 2 negative)	0.16	1.28	$1.21*10^{-95}$

Table 4: Waste degradation study: Proportionality distance between all pairs of entities (OTUs, metabolites and performance data). Clustering assignments from either PLS (first sheet, integration of OTUs and metabolites) or multiblock PLS (second sheet, integration of all three datasets). Selection with sPLS or multiblock sPLS are indicated. External file 'Table2.xls'.

Table 5: Waste degradation study: Proportionality distance for clusters identified with multiblock sparse PLS. The median distance between all pairs of profiles, within cluster and with the entire background set (outside a given cluster) is reported. A Wilcoxon test p-value assesses the difference between the medians.

cluster	median within cluster	median outside cluster	Wilcoxon test P-value
1 (comp 1 positive)	0.36	1.21	$1.25*10^{-55}$
-1 (comp 1 negative)	0.31	0.88	$9.84*10^{-36}$
2 (comp 2 positive)	0.03	0.87	$3.34*10^{-5}$
-2 (comp 2 negative)	0.27	0.73	$7.18*10^{-22}$

References

Durbán M, Harezlak J, Wand M, Carroll R. Simple fitting of subject-specific curves for longitudinal data. *Statistics in medicine* **24** (2005) 1153–1167.

Ruppert D. Selecting the number of knots for penalized splines. *Journal of computational and graphical statistics* **11** (2002) 735–757.
